# Retraction: Adaptive Neuro-Fuzzy Methodology for Noise Assessment of Wind Turbine

**DOI:** 10.1371/journal.pone.0215801

**Published:** 2019-04-23

**Authors:** The PLOS ONE Editors

Following publication, concerns have been raised regarding overlap of text between this article [1] and a number of other previously published works.

Segments of text in the Abstract, Introduction, Materials and Methods, and Conclusion sections are similar or identical to excerpts from other previously published works, some of which are cited, but it is not made clear that text has been re-used verbatim from these sources.

There is text overlap with papers from other author groups, specifically, in the Abstract [2,3], in the Introduction [4–7], in the Materials and Methods [8], and in the Conclusion [9].

The Introduction, Materials and Methods, and Conclusion sections also contain duplicate text from a 2014 article by some of the same authors [10], which has since been retracted.

In view of the extent of the overlapping text, the *PLOS ONE* Editors retract this article.

SS did not agree with retraction. DB, RH, SM did not respond. The corresponding author stands by the article as an independent contribution.
